# Humans Have Antibodies against a Plant Virus: Evidence from Tobacco Mosaic Virus

**DOI:** 10.1371/journal.pone.0060621

**Published:** 2013-04-03

**Authors:** Ruolan Liu, Radhika A. Vaishnav, Andrew M. Roberts, Robert P. Friedland

**Affiliations:** 1 Department of Neurology, University of Louisville School of Medicine, Louisville, Kentucky, United States of America; 2 Department of Physiology and Biophysics, University of Louisville School of Medicine, Louisville, Kentucky, United States of America; University of South Florida College of Medicine, United States of America

## Abstract

Tobacco mosaic virus (TMV), a widespread plant pathogen, is found in tobacco (including cigarettes and smokeless tobacco) as well as in many other plants. Plant viruses do not replicate or cause infection in humans or other mammals. This study was done to determine whether exposure to tobacco products induces an immune response to TMV in humans. Using a sandwich ELISA assay, we detected serum anti-TMV antibodies (IgG, IgG1, IgG3, IgG4, IgA, and IgM) in all subjects enrolled in the study (20 healthy smokers, 20 smokeless-tobacco users, and 20 non-smokers). Smokers had a higher level of serum anti-TMV IgG antibodies than non-smokers, while the serum level of anti-TMV IgA from smokeless tobacco users was lower than smokers and non-smokers. Using bioinformatics, we also found that the human protein TOMM40L (an outer mitochondrial membrane 40 homolog – like translocase) contains a strong homology of six contiguous amino acids to the TMV coat protein, and TOMM40L peptide exhibited cross-reactivity with anti-TMV antibodies. People who smoke cigarettes or other tobacco products experience a lower risk of developing Parkinson’s disease, but the mechanism by which this occurs is unclear. Our results showing molecular mimicry between TMV and human TOMM40L raise the question as to whether TMV has a potential role in smokers against Parkinson’s disease development. The potential mechanisms of molecular mimicry between plant viruses and human disease should be further explored.

## Introduction

Tobacco mosaic virus (TMV), the first plant virus discovered, is a single stranded RNA virus. It is present worldwide and is known to infect over 150 different plants, including tobacco, tomatoes, peppers, and cucumbers [1]. Because of its stability at high temperatures, TMV resists tobacco manufacturing processes and can be present in cigarettes, chewing tobacco and cigars for many years. To date there are no efficient chemical treatments that protect plants from virus infection. Similar to bacterial infections, plant viruses are transmissible among plants by direct contact, such as from contaminated farm tools and human hands. Unlike animal viruses, plant viruses cannot replicate in humans or other animals, largely due to the lack of specific receptors for recognition and entry into host cells. However, it has been demonstrated that cowpea mosaic virus enters the bloodstream in mice from the intestine when administrated in cowpea leaves and induces the production of antibodies without replicating [2], [3], [4]. More recently, a case-control study showed that pepper mild mottle virus may be found in human feces and is associated with clinical immune responses [5]. These studies suggest that plant viruses may play a role in human health and disease. Until now, the possible effects of consumption of TMV in tobacco products have not been investigated.

Tobacco smoking has been shown to cause cancers [6], heart disease [7], and chronic obstructive lung disease [8]. It also increases the risk for development of multiple autoimmune disorders such as rheumatoid arthritis [9] and multiple sclerosis [10], [11], [12]_ENREF_6. Although the health risks of tobacco smoking are well documented, increasing evidence suggests that smokers have a lower incidence of some inflammatory and neurodegenerative diseases. For example, smoking is reported to reduce human autoimmune responses in systemic lupus erythematosus [13] and ulcerative colitis [14]. Of particular interest to neurodegenerative disorders, epidemiological studies consistently show smokers to have a lower risk of developing Parkinson’s disease [15], [16] which is associated with a long duration of smoking rather than smoking intensity [17]. Such an inverse association is also observed in people who use chewing tobacco [18]. The protective effects of smoking have been suggested to result from the ability of nicotine (the main addictive ingredient of tobacco) to inducing immunosuppression [11] and neuroprotective action [19], [20]_ENREF_15, but the biological mechanisms by which this occurs remain largely unclear. As a complex mixture of more than 4,700 chemical compounds, many constituents of cigarettes have been shown to modulate immune function including both the humoral and cell-mediated immune responses [11]. Tobacco mosaic virus can survive for years in cigars and cigarettes made from infected tobacco leaves, and TMV can be found on the surface of cigarettes. Therefore, we presume that smokers are more likely to be exposed to TMV than non-smokers. We tested whether exposure to tobacco products induces immune responses to TMV in humans and compared the differences among individuals who were smokers, smokeless tobacco users and non-smokers. Identification of mechanisms for TMV-elicited specific immune responses may aid in defining the etiology and pathogenesis of smoking-related human diseases.

## Materials and Methods

### Ethics Statement

The study protocol for the use of human subjects was approved by the Committee of the Institutional Review Board of the University of Louisville. Written informed consent was obtained from each subject prior to enrollment according to the Declaration of Helsinki guidelines.

### Subjects

Healthy male volunteers were recruited in this study: 20 non-users of tobacco products (mean age 36.6 years, range 20–62 years), 20 current users of smokeless tobacco (mean age 39.3 years, range 22–57 years), and 20 current smokers (mean age 46.2 years, range 21–60 years). Race/ethnicity data and tobacco use history of participants were obtained from direct interview (**Table S1**).

### Preparation of serum sample

A total 10 ml of whole blood was collected into a non-anticoagulant test tube by venipuncture and left undisturbed at room temperature for 15–30 minutes, followed by centrifugation at 1,500 × g for 10 minutes to remove the clot. The resulting supernatant (serum) was immediately transferred into 1 ml aliquots and stored at –80°C until analysis.

### Virus and peptide

Tobacco mosaic virus common strain U1 (one of tobamoviruses, TMV U1,Vulgare) was kindly provided by K. E. Palmer (Large Scale Biology Corporation, Vacaville, CA) and used to determine anti-TMV antibody levels in human serum by Enzyme-linked immunosorbent assay (ELISA). The translocase of outer mitochondrial membrane 40 homolog (yeast)–like (TOMM40L) peptide (a blocking control for specificity of TOMM40L antibody) was purchased from Fitzgerald Industries International (Acton, MA).

### Measurement of anti-TMV antibodies

Serum anti-human TMV IgG antibodies and anti-human TMV IgA/D/E/M antibodies were detected by ELISA as described previously [21] with modifications. Briefly, *Nunc Immuno maxiSorp^™^* plates were coated with 100 µl/well of murine TMV monoclonal antiserum (PVAS931, 1∶100,000 dilution; American Type Culture Collection [ATCC], Manassas, VA) in coating buffer (0.1 M sodium carbonate, pH 9.5) at 4°C overnight. After blocking with 10% Fetal Bovine Serum (FBS)**,** 100 µl per well of 2.5 µg/mL TMV U1 was added and incubated for 1 h at room temperature, then serum samples were added and incubated for 2 h at room temperature. Plates were then incubated for 1 h at room temperature with horseradish peroxidase (HRP)-conjugated goat anti-human IgG, IgG1, IgG2, IgG3, IgG4, IgA, IgD, IgE or IgM (1∶1000 dilution for anti-IgG, IgG1, IgG2, IgG3, IgG4 and IgE; 1∶2000 dilution for anti-IgA; 1∶4000 dilution for anti-IgD; and 1∶10000 dilution for anti-IgM) (anti-Human IgG/IgA from BD Pharmingen, San Jose, CA; anti-human IgG subclass/ IgM from Invitrogen, Camarillo, CA; and anti-human IgD/IgE from SouthernBiotech, Birmingham, Alabama), followed by color development with TMB (3, 3', 5, 5'-tetramethylbenzidine) substrate. Results were expressed as optical density (O.D.) value at 450 nm (iMark™ microplate absorbance reader; BIO-RAD, Hercules, CA) with double or triple wells per sample. Values obtained from serum dilution buffer were subtracted from those with subject samples.

### Sequence similarity analysis

Using the TMV coat protein sequence, we performed a Basic Local Alignment Search Tool (BLAST) analysis at the National Center for Biotechnology Information (NCBI) website (http://blast.ncbi.nih.gov/Blast.cgi) to search its sequence homology with any human proteins of our potential interests. The parameters for the searches were set for maximum target sequence at 1000, expect threshold at 1000, word size at 2, matrix at PAM30 (Point Accepted Mutation 30), and no adjustments or filters included.

### Cross-reactivity of anti-TMV antibodies with TOMM40L peptide

The cross-reactivity of anti-TMV antibodies to TOMM40L peptide was determined by an Agdia Tobacco mosaic virus PathoScreen ELISA kit (Agdia, Elkhart, IN). 100 µl of TOMM40L peptide (10 µg/ml) or TMV U1 (2.5 µg/ml, as positive control) were added to an anti-TMV-coated 96-well microtiter plate and incubated for 2 h at room temperature. After washing, the plates were further incubated with alkaline phosphatase-conjugated enzyme for 2 h and then developed with p-Nitrophenyl (PNP) substrate. Results were expressed as O.D. at 405 nm.

### Statistical analysis

Results are presented as means ± SE, and statistical analysis was done using GraphPad Prism software (GraphPad Software, Inc., San Diego, CA). An unpaired Student’s *t* test or Mann-Whitney *U* test was used for two group comparisons. One-way analysis of variance (ANOVA) with Tukey post-hoc test or Kruskal-Wallis nonparametric test with Dunn post-hoc test was used for multiple comparisons among groups. *p* <0.05 was considered statistically significant.

## Results

### Anti-TMV IgG antibodies are present in human serum

**Figure 1:**
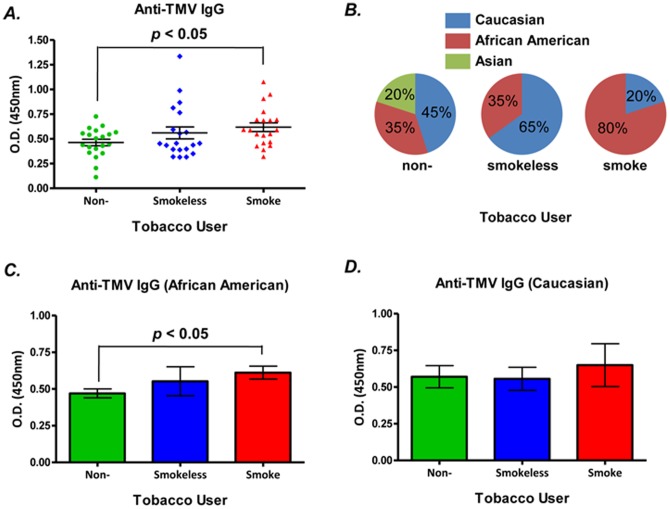
Effects of tobacco-smoke and smokeless-tobacco exposure on serum anti-Tobacco Mosaic Virus (TMV) IgG. Sera were obtained from groups of smoke tobacco users (smokers), smokeless tobacco users (tobacco chewers) and non-tobacco users (n  =  20/group). Serum anti-TMV IgG levels were measured by a customized sandwich ELISA assay. The serum dilution factor was 1∶100 and the data is expressed as O.D. value (mean ± SE). All data are representative of two to three assays with similar results. (A) Serum anti-TMV IgG in whole study population; (B) Ethnic characteristics of the study population; (C) Serum anti-TMV IgG in African American study population; and (D) Serum anti-TMV IgG in Caucasian study population. *p*-value, Student’s *t* test, *p < 0.05.

All 60 subjects enrolled in the study had serum IgGs that reacted with TMV (**Figure 1**). The level of anti-TMV IgG was higher in smokers than non-users (**Figure 1A**: 0.62 ± 0.04 vs 0.46 ± 0.03; *p* < 0.01, *t* test). The levels of anti-TMV IgG did not differ between smokers and smokeless tobacco users and between smokeless tobacco users and non-users (**Figure 1A**).

As human IgG has four subclasses (IgG1, 2, 3 and 4) named in order of their abundance in serum, TMV-specific IgG subclasses were analyzed. Anti-IgG1, 3, and 4 were all present in human sera whereas very low seropositivity was detected for serum anti-TMV IgG2. Anti-IgG1 was the predominant subclass and the only one that smokers had a significantly higher level than non-smokers (**Table 1**). The levels of anti-IgG1, 3, and 4 were not significantly different among smokeless-tobacco users and non-smokers (**Table 1**).

**Table 1:** Levels of Anti-TMV IgG Subclass in Human Serum.

Anti-TMV Abs	Non-smokers	Smokers	*p*-value^a^
IgG1	1.71 ± 0.04	1.84 ± 0.03	0.02*
IgG2	0.08 ± 0.01	0.09 ± 0.01	0.24
IgG3	0.31 ± 0.02	0.36 ± 0.03	0.16
IgG4	0.36 ± 0.03	0.41 ± 0.03	0.20

Sera were obtained from groups of smokers and non-tobacco users (n = 20/group). Serum anti-TMV IgG subclass levels were measured by a customized sandwich ELISA assay. The serum dilution factor for anti-TMV IgG1/IgG3/IgG4 was 1∶100 and for anti-TMV-IgG2 was 1∶10. Data represent two to three independent experiments with double wells per subject. Results are expressed as O.D. values (mean ± SE); *p*-value, ^a^ Student’s *t* test, *p < 0.05.

To determine if ethnic differences among enrolled groups were associated with TMV immune responses, the 60 subjects was stratified into Asian, Black/African American and White/Caucasian ethnic groups. Asian male subjects were found only in the non-user group, thus we focused on African American and Caucasian groups in our subsequent analysis (**Figure 1B**). Elevated serum levels of anti-TMV IgG were observed in the African-American smokers (**Figure 1C:** 0.61 ± 0.04 vs 0.47 ± 0.03; *p* < 0.05, *t* test), but not in the Caucasian smokers (**Figure 1D:** 0.65 ± 0.15 vs 0.57 ± 0.08; *p*  =  0.05, *t* test). For the Caucasian ethnic group, the serum level of anti-TMV IgG1 from smokers was marginally higher than non-smokers (**Table S2**). However, among the African-American group, the levels of anti-TMV IgG3 and IgG4, but not IgG1, were significantly higher in smokers than non-smokers (**Table S3**).

### Anti-TMV IgM, IgA, IgD, and IgE levels in human serum

**Figure 2:**
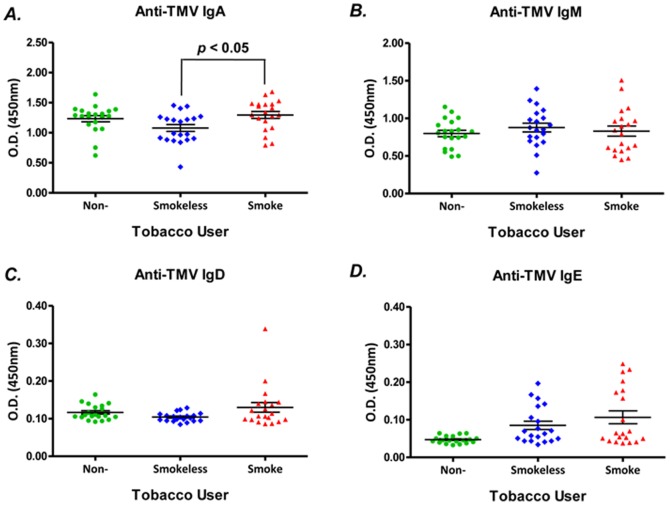
Effects of tobacco-smoke and smokeless-tobacco exposure on serum anti-TMV IgA, IgM, IgD and IgE. Sera were obtained from groups of smokers, smokeless tobacco users and non-tobacco users (n = 20/group). Serum anti-TMV IgA, IgM, IgD, and IgE levels were measured by a customized sandwich ELISA assay. The serum dilution factors were 1∶50 for anti-TMV IgA and IgM and 1∶5 for anti-TMV IgD and IgE. Data is expressed as O.D. values (mean ± SE). All data are representative of two to three assays with similar results. (A) Serum anti-TMV IgA in whole study population; (B) Serum anti-TMV IgM in whole study population; (C) Serum anti-TMV IgD in whole study population; (D) Serum anti-TMV IgG in whole study population. *p*-value, ANOVA, *p < 0.05.

In addition to anti-IgGs, the other four classes of human antibodies, namely IgM, IgA, IgD, and IgE, were evaluated in this study (**Figure 2**). Anti-TMV IgM and IgA were detected in serum from all three groups. It is interesting to note that a significantly reduced level of serum anti-TMV IgA was found in tobacco chewers compared to smokers (1.08 ± 0.06 vs 1.30 ± 0.06; *p* < 0.05, ANOVA). Although it was also lower, it was not significantly different from non-smokers (1.08 ± 0.06 vs 1.23 ± 0.05; *p* > 0.05, ANOVA) (**Figure 2A**). The levels of anti-TMV IgM were not different among these three groups (smokers: 0.83 ± 0.07, tobacco chewers: 0.88 ± 0.06, non-users: 0.80 ± 0.04; *p* > 0.05, ANOVA) (**Figure 2B**). Levels of anti-TMV IgD and anti-TMV IgE were hardly detectable in serum. Only one person (a smoker) showed a high seropositivity (1/20; **Figure 2C**); and, approximately, 25% (5/20) of smokeless tobacco uers and 35% (7/20) of smokers had a detectable low level of anti-TMV IgE in their sera (**Figure 2D**).

### Anti-TMV antibody specific cross-react with TOMM40L protein

**Figure 3:**
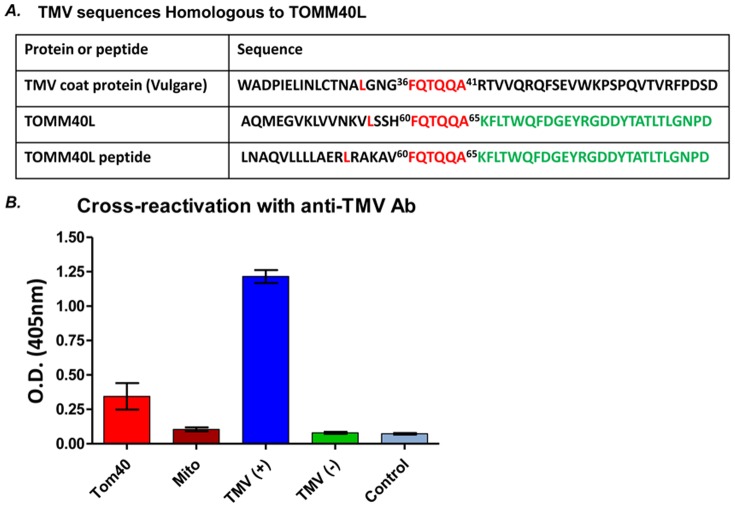
Cross reactivity between TMV and TOMM40L. (A) Sequence homology comparison among TMV-Vulgare coat protein. Basic Local Alignment Search Tool (BLAST) indicated the regions of local sequence similarity (color red) among TMV and TOMM40L gene and TOMM40L peptide; and sequence similarity (color green) between TOMM40L gene and peptide. (B) Cross-reactivity of anti-TMV antibodies and TOMM40L peptide determined by tobacco mosaic virus PathoScreen ELISA kit. Data are representative of two independent experiments with similar results. Results were expressed as O.D. values (mean ± SE, triple wells per group). Tom40  =  TOMM40L peptide, Mito  =  crude mitochondria pellet, TMV (+)  =  TMV-infected tobacco leaf, TMV (-)  =  TMV-negative alstroemeria leaf, and control  =  ELISA assay buffer.

Using BLAST analysis, we found that a human TOMM40L protein (-^56^L---^60^FQTQQA^65^-) showed significant sequence similarity to the TMV coat protein (-^32^L---^36^FQTQQA^41^-) with an overlap of 6 contiguous amino acids (**Figure 3A**). We also demonstrated that anti-TMV antibody cross-reacts with the TOMM40L peptide (**Figure 3B**, red bar labeled as Tom40) and reacted with TMV from TMV infected tobacco leaf (**Figure 3B**, blue bar labeled as TMV+, a positive control of the assay), but did not react to crude mitochondrial pellet (**Figure 3B**, brown bar labeled as Mito), TMV-negative Alstroemeria leaf (**Figure 3B**, green bar labeled as TMV-), or buffer (**Figure 3B**, light blue bar labeled Control). These results demonstrate a novel cross-reactivity of TMV antibodies to a human mitochondrial protein, TOMM40L.

## Discussion

To our knowledge, this is the first report of the presence of anti-TMV antibodies in human serum. We also report an association between tobacco smoking and anti-TMV antibodies. Smokers had higher serum antibody responses to TMV than non-tobacco users, although non-users were also seropositive. We further discovered that anti-TMV antibody cross-reacted with the human protein TOMM40L, a mitochondrial outer membrane subunit containing a strong amino acid homology to TMV coat protein.

Specific immune responses are elicited by pathogenic antigens present in viruses, and it is generally thought that they are not elicited by plant viruses due to their inability to replicate in animals [5], [22]. Plant viruses can be routinely found in foods we eat and in human feces but they are not thought to be involved in human health or disease [5]. TMV is the best characterized plant viral pathogen and almost all of its coat protein gene sequence has been shown to be antigenic [23]. TMV is widely utilized as plant virus expression vectors for vaccine antigens and biopharmaceutical protein production, but limited data are available for humans regarding immune responses elicited directly by TMV virions. A report from 1968 failed to detect serum antibodies to TMV in patients with pulmonary diseases or healthy individuals using a plant virus hemagglutination test [24]_ENREF_5. By using a modified Sandwich ELISA assay, our study shows that antibodies against TMV are present in serum of all subjects, including healthy smokers as well as non-smokers. These anti-TMV antibodies are mainly IgG, the most abundant pathogen-binding antibody in human serum (response level: IgG1>IgG4>IgG3). The four human IgG subclasses, IgG1 to IgG4, differ in their affinities for binding and in their abilities to induce effector responses [25]. Similar to most published serum antibody reports, IgG1 was found to provide the dominant response to TMV. Smokers had a significantly higher level of serum IgG (IgG1, IgG3 and IgG4) antibodies against TMV than non-smokers, while anti-TMV IgG (IgG1, IgG3 and IgG4) levels of smokeless tobacco users did not significantly differ from those of non-smokers. The increased production of anti-TMV antibodies in tobacco smokers may reflect humoral immune abnormalities or increased exposure.

At least 13 continuous antigenic determinants (epitopes) have been mapped at the surface of TMV coat protein and their structure has been extensively studied [23], [26], [27], [28], [29], including epitopes p34–39 and p28–42 that are shown to have antigenic activity [27], [29]. In the present study, we observed a striking sequence homology (6 continuous residues) between TMV coat protein epitope p36–41 and human TOMM40L protein p60–65. TOMM40L (also known as TOMM40B) is a potential channel-forming protein implicated in the importation of protein precursors into mitochondria. It forms part of the preprotein translocase of the outer mitochondrial membrane (TOM complex) containing TOMM22, TOMM40, TOMM40L and TOMM70 and interacts with mitochondrial targeting sequences [30]. In humans, a polymorphism encoding this gene complex (TOMM40) has been associated with an increased risk of developing late-onset Alzheimer disease (AD) [31], although recent results conflict with several reports [32]. Kimura et al [33] has reported that serum anti-TOMM40 antibodies occur twice as often in people with AD than in healthy subjects or in patients with multiple sclerosis. Given that mitochondrial dysfunction is a common observation in Alzheimer disease, Parkinson’s disease (PD) and other neurodegenerative disorders [34], the TOM complex may very well be involved in the etiology of neurodegenerative disorders.

Our data demonstrate that anti-TMV antibodies can cross-react with TOMM40L. These observations suggest molecular mimicry between TMV and human TOMM40L protein. Molecular mimicry is a concept by which autoimmune diseases may be caused by the structural similarity of antigenic determinants. For example, an immune response against a viral sequence shared by the host and the virus can evoke a tissue-specific immune response that is capable of eliciting cell and tissue destruction [35], [36]. Based on our findings, we hypothesize a role of anti-TMV antibody in TOMM40L-mediated human health and disease.

Parkinson’s disease is the second most common neurodegenerative disorder in the U.S. About 50,000 American are diagnosed with PD each year, with more than half a million Americans affected. Epidemiologic evidence has long suggested a negative association between smoking and the risk of PD. Statistics show that PD appears to affect men at a slightly higher rate than women and is less likely to affect African Americans or Asians than whites [37]. Scientists have not been able to explain this apparent lower incidence in certain populations. In our study, the significant increase of serum anti-TMV IgG in smokers was only found in African Americans but not in Caucasians, pointing out a possibility that the high immune response of TMV in African American smokers may be associated with a lower incidence of PD. Mitochondrial dysfunction has been widely implicated in the pathogenesis of PD [34], [38]. The PINK1 gene products which cause autosomal recessive familial PD are localized to mitochondria in several tissues [39] and a fraction of alpha-synuclein (a major player in the pathogenesis of PD) is identified in mitochondria [40]. Therefore, we speculate that the infectious agent TMV can be a driver of molecular mimicry, eliciting anti-TMV antibodies cross-reacting with TOMM40L protein to alter the host’s mitochondrial function; in smokers, anti-TMV antibodies may modulate the acquired mitochondrial defects through molecular mimicry to prevent PD development.

Molecular mimicry has been linked to a number of naturally occurring human diseases such as myasthenia gravis (the acetylcholine receptor AChRα-subunit amino acids 160–167 shows cross-reactivity with a shared homologous domain on herpes simplex virus glycoprotein D p286–293) [41] and multiple sclerosis (the myelin basic protein p85–99-specific T cell clones are activated by viral mimicry peptides) [42]. In the case of the observed sequence homology between TMV coat protein epitope 36–41 and human TOMM40L protein p60–65, it may be that anti-TMV antibodies influence mitochondrial function to alter disease processes. Although it might be expected that antibodies to mitochondrial proteins will not have access to the intracellular space, it should be noted that antinuclear antibodies are known to play an important part in autoimmune diseases including systemic lupus erythematosis and hepatitis C [43], [44]. The alteration of mitochondrial function induced by the TMV related molecular mechanism we have discussed may be secondary to cytotoxicity (and the disruption of cellular integrity) caused by amyloid aggregates, including out-of-register Beta–sheets as recently reported by Liu et al [45]. It may also be the case that anti-TMV antibodies cross-react with other proteins important in neurodegenerative disorders. The molecular mimicry process we describe here may be primarily in the CNS or may be a systemic mechanism, as alpha-synuclein is also expressed in lymphocytes [46]. Furthermore, the mechanisms of molecular mimicry we discuss could take place in microglia, which are believed to play a role in PD. Impaired mitochondrial metabolism in microglia will impair neuroinflammation and modify risk. We acknowledge that the specific influence of the molecular mimicry we report on mitochondrial function is difficult to predict. It may be that abnormalities in mitochondrial processes in PD are improved by the antibody interactions. A protective role for antibodies against a plant virus (potato virus Y) in the development of Alzheimer’s disease has been proposed [47]. It is also possible that the presence of anti-TMV antibodies elicits immune responses in the brain involving toll like receptors or other immune response elements involved in the pathogenesis of PD [48].

Our study has a number of limitations. The study’s sample size is small, with only 20 people in each group, with uneven distribution of African American subjects. The measurement of anti-TMV antibody in serum by ELISA is semi-quantitative, and we were unable to quantitatively measure antibody levels. The study population included relatively young subjects and aged adults are at higher risk for PD, so we are unable to draw a firm conclusion regarding PD. Further studies are needed to evaluate the relationships among anti-TMV and anti-TOMM40L antibodies and the development of PD, and to determine the role of TOMM40L in pathogenesis of PD. Future study of the effects of TMV exposure on human health and disease may provide a possible therapeutic strategy against Parkinson’s disease. Finally, TMV is being used as a vector for antigen delivery in bioengineered vaccines [49]. Our data suggests that immune responses to TMV antigens deserve investigation because of their potential to interfere with vaccine biodistribution or cellular metabolism.

## Supporting Information

Table S1 **History of Tobacco Use.** Tobacco use history of participants was obtained from direct interview. NA indicates data Not Available, ^a^ mean ± SE, and ^b^ percentage.
(DOCX)

Table S2 **Levels of Anti-TMV IgG Subclass in Human Serum (Caucasian).** Sera were obtained from Caucasian study population of smokers and non-tobacco users. Serum anti-TMV IgG subclass levels were measured by a customized sandwich ELISA assay. The serum dilution factor for anti-TMV IgG1/ IgG3/IgG4 was 1∶100 and for anti-TMV-IgG2 was 1∶10. Data represent two to three independent experiments with double wells per subject. Results are expressed as O.D. values (mean ± SE); *p*-value, ^a^ Student’s *t* test, *p < 0.05.
(DOCX)

Table S3 **Levels of Anti-TMV IgG Subclass in Human Serum (African American).** Sera were obtained from African American study population of smokers and non-tobacco users. Serum anti-TMV IgG subclass levels were measured by a customized sandwich ELISA assay. The serum dilution factor for anti-TMV IgG1/ IgG3/IgG4 was 1∶100 and for anti-TMV-IgG2 was 1∶10. Data represent two to three independent experiments with double wells per subject. Results are expressed as O.D. values (mean ± SE); *p*-value, ^a^ Student’s *t* test, ^b^ Mann-Whitney *U* test, *p < 0.05.
(DOCX)
