# Supplementary material for: Humans Have Antibodies against a Plant Virus: Evidence from Tobacco Mosaic Virus
Source: PLoS One. 2013 Apr 3;8(4):e60621. doi: 10.1371/journal.pone.0060621 (PMC3615994; doi:10.1371/journal.pone.0060621)
Supplement: Table S1 — History of Tobacco Use. Tobacco use history of participants was obtained from direct interview. NA indicates data Not Available, a mean ± SE, and b percentage. (DOCX) [file pone.0060621.s001.docx]

**Table S1**

| **Study Groups** | **Non-User**  **(n = 20)** | **Smokeless Tobacco User**  **(n = 20)** | **Smoke User**  **(n = 20)** |
| --- | --- | --- | --- |
| **Cigarette Use** |  |  |  |
| Age started (years)^a^ | NA | 17.9 ± 1.6 | 18.9 ± 4.3 |
| **Current use ^b^** | 0.0% | 30.0% | 100.0% |
| Duration (years) ^a^ | NA | NA | 27.3 ± 6.3 |
| Frequency (pack/day) ^a^ | NA | NA | 1.1 ± 0.2 |
| Past use ^b^ | 10.0% | 70.0% | NA |
| **Smokeless Tobacco Use** |  |  |  |
| Age started ^a^ | NA | 18.7 ± 1.5 | NA |
| Duration (years) ^a^ | NA | 20.6 ± 2.5 | NA |
| Frequency (can/day) ^a^ | NA | 0.8 ± 0.1 | NA |
| **Worked with Tobacco Products** ^b^ | 0.0% | NA | 30.0% |
