# Supplementary material for: Humans Have Antibodies against a Plant Virus: Evidence from Tobacco Mosaic Virus
Source: PLoS One. 2013 Apr 3;8(4):e60621. doi: 10.1371/journal.pone.0060621 (PMC3615994; doi:10.1371/journal.pone.0060621)
Supplement: Table S2 — Levels of Anti-TMV IgG Subclass in Human Serum (Caucasian). Sera were obtained from Caucasian study population of smokers and non-tobacco users. Serum anti-TMV IgG subclass levels were measured by a customized sandwich ELISA assay. The serum dilution factor for anti-TMV IgG1/ IgG3/IgG4 was 1∶100 and for anti-TMV-IgG2 was 1∶10. Data represent two to three independent experiments with double wells per subject. Results are expressed as O.D. values (mean ± SE); p-value, a Student’s t test, *p < 0.05. (DOCX) [file pone.0060621.s002.docx]

**Table S2**

| **Anti-TMV** **Abs** | **Non-smokers** | **Smokers** | ***P*-value^a^** |
| --- | --- | --- | --- |
| **IgG1** | **1.58 ± 0.06** | **1.84 ± 0.16** | **0.06** |
| **IgG2** | **0.09 ± 0.01** | **0.09 ± 0.01** | **0.44** |
| **IgG3** | **0.33 ± 0.04** | **0.37 ± 0.10** | **0.31** |
| **IgG4** | **0.38 ± 0.04** | **0.42 ± 0.11** | **0.31** |
