# Supplementary material for: Humans Have Antibodies against a Plant Virus: Evidence from Tobacco Mosaic Virus
Source: PLoS One. 2013 Apr 3;8(4):e60621. doi: 10.1371/journal.pone.0060621 (PMC3615994; doi:10.1371/journal.pone.0060621)
Supplement: Table S3 — Levels of Anti-TMV IgG Subclass in Human Serum (African American). Sera were obtained from African American study population of smokers and non-tobacco users. Serum anti-TMV IgG subclass levels were measured by a customized sandwich ELISA assay. The serum dilution factor for anti-TMV IgG1/ IgG3/IgG4 was 1∶100 and for anti-TMV-IgG2 was 1∶10. Data represent two to three independent experiments with double wells per subject. Results are expressed as O.D. values (mean ± SE); p-value, a Student’s t test, b Mann-Whitney U test, *p < 0.05. (DOCX) [file pone.0060621.s003.docx]

**Table S3**

| **Anti-TMV** **Abs** | **Non- smokers** | **Smokers** | ***P*-value** |
| --- | --- | --- | --- |
| **IgG1** | **1.78 ± 0.05** | **1.84 ± 0.03** | **0.11^a^** |
| **IgG2** | **0.08 ± 0.01** | **0.09 ± 0.01** | **0.17^a^** |
| **IgG3** | **0.26 ± 0.01** | **0.36 ± 0.03** | **0.007^b^*** |
| **IgG4** | **0.30 ± 0.02** | **0.41 ± 0.03** | **0.007^b^*** |
